# Maternal High Fat Feeding Does Not Have Long-Lasting Effects on Body Composition and Bone Health in Female and Male Wistar Rat Offspring at Young Adulthood

**DOI:** 10.3390/molecules181215094

**Published:** 2013-12-06

**Authors:** Paula M. Miotto, Laura M. Castelli, Foyinsola Amoye, Paul J. LeBlanc, Sandra J. Peters, Brian D. Roy, Wendy E. Ward

**Affiliations:** 1 Center for Bone and Muscle Health, Brock University, 500 Glenridge Avenue, St. Catharines, ON, L2S 3A1, Canada; 2 Department of Kinesiology, Faculty of Applied Health Sciences, Brock University, 500 Glenridge Avenue, St. Catharines, ON, L2S 3A1, Canada; 3 Department of Community Health Sciences, Faculty of Applied Health Sciences, Brock University, 500 Glenridge Avenue, St. Catharines, ON, L2S 3A1, Canada

**Keywords:** bone mineral, bone strength, fatty acids, high fat diet, nutritional programming, rats

## Abstract

High fat diets adversely affect body composition, bone mineral and strength, and alter bone fatty acid composition. It is unclear if maternal high fat (HF) feeding permanently alters offspring body composition and bone health. Female rats were fed control (CON) or HF diet for 10 weeks, bred, and continued their diets throughout pregnancy and lactation. Male and female offspring were studied at weaning and 3 months, following consumption of CON diet. At weaning, but not 3 months of age, male and female offspring from dams fed HF diet had lower lean mass and higher fat and bone mass, and higher femur bone mineral density (females only) than offspring of dams fed CON diet. Male and female offspring femurs from dams fed HF diet had higher monounsaturates and lower *n*6 polyunsaturates at weaning than offspring from dams fed CON diet, where females from dams fed HF diet had higher saturates and lower *n*6 polyunsaturates at 3 months of age. There were no differences in strength of femurs or lumbar vertebrae at 3 months of age in either male or female offspring. In conclusion, maternal HF feeding did not permanently affect body composition and bone health at young adulthood in offspring.

## 1. Introduction

Long-term high fat consumption has been shown to alter body composition and bone health (*i.e.*, bone quantity, strength, and fatty acid composition) in male and female rodents. These feeding studies have generally started when rodents are young adults (post-weaning) and for varying durations, such as 8 to 28 weeks [1,2,3,4,5,6,7,8,9]. Furthermore, some of these studies did not adjust for the level of protein, vitamins and minerals within the respective diets [1,2,4,6,7,8,9]. This would ensure that effects shown in body composition and bone health are due to the elevated dietary fat content and not lower levels of nutrients per energy basis. These studies have demonstrated greater fat mass [1,10] and lower lean [8,11] and bone mass [7,9] following high fat feeding. Bone quantity in terms of bone mineral content (BMC) and density (BMD) has been observed to be lower in excised femurs and lumbar vertebrae as a result of high fat feeding [5,6]. Moreover, bone strength—a surrogate measure of fracture risk has been reported to be reduced with high fat feeding [5]. Whether this corresponds with altered bone fatty acid composition is not well understood, as it has been shown that the skeleton is responsive to changes in dietary fat. For example, the fatty acid compositions of femurs and vertebrae have been shown to reflect the quantity and composition of fatty acids in the diet. As such, rats fed a high saturated or polyunsaturated fat diet had greater total saturated fat and polyunsaturated fat in bone, respectively [3,12,13,14,15]. Overall, high fat feeding has demonstrated adverse effects on body composition, bone mineral and strength, and has altered bone fatty acid content.

Whether maternal high fat feeding can permanently impact offspring body composition and bone health across the lifespan is less understood. *In utero* and suckling exposure to a high fat diet may result in nutritional programming—a concept that refers to a permanent effect of a food or food component on a tissue or a system, when exposure occurs during early life. Previous literature has shown that maternal high fat feeding resulted in greater fat mass and lower lean mass [1,16,17] in male and female offspring early in life (from birth to weaning). However, whether these differences persist into adulthood (e.g., 3 months of age) following the consumption of a balanced control diet requires further study. Less information is available regarding the effects of maternal high fat feeding and offspring bone quantity, strength, and fatty acid composition. For instance, Lanham, *et al.* [18] found that maternal high fat feeding (18% lard by weight; level of proteins, vitamins and minerals were adjusted for elevated fat content) resulted in higher bone density and lower bone strength in femurs from male but not female offspring at 7.5 months of age. However, since offspring were weaned to a high fat diet rather than control diet, it is unclear if the effects were due to nutritional programming. In contrast, Devlin, *et al.* [19] reported no differences in femoral strength from male and female offspring (3.5 and 6.5 months of age) as a result of maternal high fat feeding (23% fat by weight with 20% lard and 3% soybean oil; level of protein, vitamins and minerals were adjusted for elevated fat content). Despite this finding, it is unclear whether maternal high fat feeding corresponded to lower femoral bone mineral, or if vertebrae were similarly affected, both in terms of bone mineral and strength. In regard to bone fatty acid composition, there has been no investigation to determine if maternal diet “programs” offspring fatty acid composition or amount.

Overall, the effects of maternal high fat feeding on offspring body composition and bone health have focused on offspring at earlier stages of life or during adulthood. Examination of offspring at both weaning and adulthood—after being weaned onto a control diet may be important to determine whether changes evident in adulthood are a direct result of nutritional programming. Moreover, measurement of bone fatty acid composition would elucidate how responsive offspring bone is to mother’s diet at weaning, and if differences in fatty acid composition persist at young adulthood. Thus, the purpose of this study was to determine whether maternal high fat feeding contributes to altered body composition (lower lean and bone mass, higher fat mass), lower bone mineral, and altered fatty acid composition in male and female offspring at weaning, and if these effects persist and correspond with lower bone strength at 3 months of age after consuming a control diet post-weaning.

## 2. Results

### 2.1. Litter Characteristics

There were no differences in litter size (n = 14–15), the ratio of females to males in a litter (females, n = 7; males, n = 7), or litter weight at PND 3 between groups. However, at PND 12, offspring in litters from dams fed HF diet weighed significantly more than offspring from dams fed CON diet (438 ± 14 g and 386 ± 6 g, respectively).

### 2.2. Effect of High Fat Feeding in Dams

#### 2.2.1. Body Weight

There were no differences in body weight at time of breeding, however, final body weight of dams fed HF diet were significantly heavier than dams fed CON diet at the end of lactation (Table 1). Dams fed HF diet had significantly higher energy intake than dams fed CON diet for all but two out of the 10 weeks that food intake was measured, from the time of arrival through to the start of breeding (data not shown). It was not possible to accurately measure food intake of dams during breeding due to the presence of the male, or during pregnancy and lactation to minimize cage disturbances.

#### 2.2.2. Plasma Hormone Concentrations

There were no differences in plasma concentrations of leptin, IL-6, MCP-1, 17β-estradiol and progesterone between dams fed HF *versus* CON diet (Table 1). No comparison was made for plasma TNF-α as some rats had levels below the level of detection for the assay. However, more dams fed HF diet (n = 5) had detectable levels than dams fed CON diet (n = 2).

**Table 1:** Body weight, plasma hormones, and bone outcomes of dams.

Outcome	Treatment		*p* Value
	CON	HF	Diet
**Body Weight**			
Time of breeding (g)	330 ± 9	343 ± 12	0.412
Final body weight (g)	324 ± 5	350 ± 10	0.028
**Plasma Hormones**			
Leptin (pg/mL)	1357.7 ± 158.5	1936.8 ± 331.8	0.135
IL-6 (pg/mL)	227.7 ± 63.1	506.3 ± 122.4	0.130
TNF-α (pg/mL)	BD	BD	
MCP-1 (pg/mL)	391.2 ± 122.8	475.2 ± 124.9	0.525
17β-estradiol (ng/mL)	0.03 ± 0.01	0.04 ± 0.01	0.485
Progesterone (ng/mL)	21.5 ± 3.4	29.4 ± 3.2	0.110
**Bone Mineral**			
*Whole femur*			
BMC (mg)	355.9 ± 15.4	367.0 ± 13.4	0.584
BMD (mg/mm^2^)	1.70 ± 0.05	1.76 ± 0.04	0.379
*1/3 Proximal femur*			
BMC (mg)	122.0 ± 6.2	123.9 ± 4.6	0.800
BMD (mg/mm^2^)	1.62 ± 0.05	1.69 ± 0.05	0.332
*LV1-3*			
BMC (mg)	244.5 *±* 15.6	249.5 ± 11.5	0.792
BMD (mg/mm^2^)	1.45 ± 0.05	1.48 ± 0.04	0.625
**Bone Strength**			
*Femur midpoint*			
Peak load (N)	125.4 ± 9.1	138.3 ± 7.1	0.948
*Femur neck*			
Peak load (N)	82.9 ± 17.4	62.3 ± 8.2	0.300
*LV3*			
Peak load (N)	188.2 ± 18.4	220.4 ± 17.2	0.219

Notes: Data is expressed as mean ± SEM (n = 9 per group). BD, values below the level of detection, no comparison made.

#### 2.2.3. Bone Mineral and Strength in Femurs and Lumbar Vertebrae

There were no differences in BMC or BMD of whole femurs, at the 1/3 proximal site of femurs or LV1-3 between groups. There were also no differences in peak load at the femur midpoint, femur neck, or LV3 between groups (Table 1).

#### 2.2.4. Fatty Acid Composition of Femurs

Dams that consumed HF diet had higher percent mole fraction of MUFAs, as well as lower *n*3 and *n*6 PUFAs compared to dams fed CON diet. There were no differences in percent mole fraction of SFAs between dams fed CON and HF diet (Table 2).

**Table 2:** Femur fatty acid composition of dams.

Fatty acid	Treatment		*p* Value
	CON	HF	Diet
14:0	1.66 ± 0.10	1.62 ± 0.04	0.750
16:0	24.97 ± 0.89	25.07 ± 0.34	0.917
18:0	7.94 ± 0.26	9.53 ± 0.27	<0.001
16:1	4.47 ± 0.25	3.88 ± 0.11	0.068
18:1	25.52 ± 0.92	38.31 ± 0.55	<0.001
18:3*n*3	1.85 ± 0.09	0.39 ± 0.02	<0.001
20:3*n*3	3.42 ± 0.23	3.35 ± 0.42	0.884
18:2*n*6	25.09 ± 0.71	12.84 ± 0.15	<0.001
Total SFAs	36.02 ± 1.26	37.76 ± 0.57	0.251
Total MUFAs	31.10 ± 0.85	43.58 ± 0.61	<0.001
Total PUFAs	32.89 ± 0.71	18.66 ± 0.70	<0.001
*n*3 PUFAs	6.42 ± 0.21	4.22 ± 0.46	<0.001
*n*6 PUFAs	26.47 ± 0.69	14.44 ± 0.27	<0.001

Notes: Data is expressed as mean ± SEM (n = 7 or 8 per group) for percent mole fraction of femur total fatty acids. Percent mole fraction of fatty acids below 1% are not shown.

### 2.3. Effect of Maternal Diet in Female Offspring

#### 2.3.1. Body Weight and Body Composition

**Figure 1:**
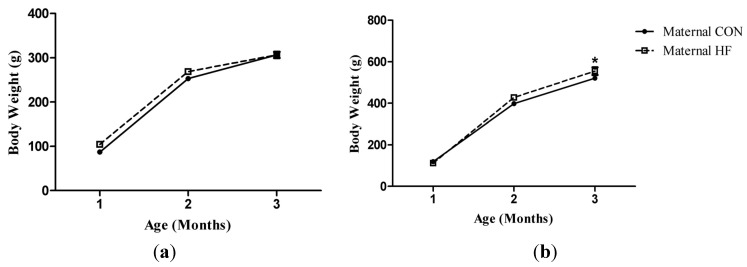
Female (**a**) and male (**b**) offspring body weight growth curves from dams fed CON or HF diet. * Maternal HF > Maternal CON, *p* < 0.05.

There were no differences in body weight from 1 to 3 months of age in offspring from dams fed HF or CON diets (Figure 1a). There were significant interactions for age and maternal diet when examining % lean mass, % fat mass, and % bone mass. Weanling offspring from dams fed HF diet had lower lean mass and higher fat mass than weanling offspring from dams fed CON diet. Furthermore, weanling offspring from dams fed CON diet had greater lean mass and lower fat mass than at 3 months of age. At weaning, offspring from dams fed HF diet had greater % bone mass than offspring from dams fed CON diet. Offspring from dams fed HF diet had lower % bone mass at 3 months of age than at weaning. Offspring from dams fed CON diet had higher % bone mass at 3 months of age than at weaning (Table 3).

#### 2.3.2. Plasma Hormone Concentrations

There was a significant interaction of age and maternal diet for MCP-1, where weanling offspring from dams fed HF diet had greater concentrations than weanling offspring from dams fed CON diet. Furthermore, weanling offspring from dams fed HF diet had greater MCP-1 levels than at 3 months of age.

**Table 3:** Female offspring body composition, plasma hormones, and bone outcomes.

Outcomes	Weaning		3 Months		*p* Values		
	Maternal CON	Maternal HF	Maternal CON	Maternal HF	Age x Diet	Age	Diet
**Body Composition**							
% Lean mass	85.6 ± 1.1	70.5 ± 2.2 *^a^*	72.6 ± 3.6 *^a^*	73.0 ± 3.1	0.013	0.079	0.017
% Fat mass	13.1 ± 1.1	27.7 ± 2.1 *^a^*	25.9 ± 3.5 *^a^*	25.4 ± 3.1	0.014	0.076	0.020
% Bone mass	1.29 ± 0.06	1.82 ± 0.09 *^a^*	1.54 ± 0.05 *^a^*	1.62 ± 0.01 *^b^*	0.001	0.725	<0.001
**Plasma Hormones**							
Leptin (pg/mL)	14343 ± 4102	12858 ± 6909	2763 ± 190	2678 ± 372	0.707	<0.001	0.685
IL-6 (pg/mL)	77 ± 16	159 ± 46	313 ± 56	305 ± 36	0.281	<0.001	0.380
TNF-α (pg/mL)	BD	BD	10.1 ± 1.6	10.2 ± 1.2			0.965
MCP-1 (pg/mL)	282 ± 47	616 ± 149 *^a^*	293 ± 52	235 ± 36 *^b^*	0.026	0.036	0.113
17β-estradiol (ng/mL)	NM	NM	0.04 ± 0.01	0.05 ± 0.02			0.265
Progesterone (ng/mL)	NM	NM	29.5 ± 5.9	19.4 ± 3.1			0.146
**Bone Mineral**							
*Whole femur*							
BMC (mg)	17.0 ± 1.5	23.1 ± 1.9	373.6 ± 13.6	370.4 ± 13.6	0.642	<0.001	0.856
BMD (mg/mm^2^)	0.35 ± 0.01	0.41 ± 0.02 *^a^*	1.97 ± 0.02 *^a^*	1.96 ± 0.02 *^b^*	0.040	<0.001	0.382
*1/3 Proximal femur*							
BMC (mg)	5.1 ± 0.4	6.5 ± 0.5	129.9 ± 4.9	133.9 ± 2.0	0.737	<0.001	0.384
BMD (mg/mm^2^)	0.37 ± 0.01	0.42 ± 0.02	2.00 ± 0.03	2.01 ± 0.03	0.221	<0.001	0.475
*LV1-3*							
BMC (mg)	12.6 ± 1.0	16.1 ± 1.1	301.7 ± 14.9	306.9 ± 7.9	0.913	<0.001	0.595
BMD (mg/mm^2^)	0.33 ± 0.02	0.37 ± 0.02	1.91 ± 0.05	1.92 ± 0.03	0.640	<0.001	0.435
**Bone Strength**							
*Femur midpoint*							
Peak load (N)	NM	NM	144.4 ± 8.5	138.2 ± 6.0			0.114
*Femur neck*							
Peak load (N)	NM	NM	84.9 ± 4.3	78.3 ± 2.0			0.600
*LV3*							
Peak load (N)	NM	NM	380.7 ± 23.8	360.3 ± 19.9			0.523

Notes: Data is expressed as mean ± SEM ((n = 9 per group); except for measures of body composition at weaning (n = 7/group), leptin (n = 6/group; 3 values per group exceeded highest standard), femoral BMC and BMD at weaning (n = 7 in offspring from dams fed CON as some bones were fractured upon extraction), and LV3 peak load (n = 7/group as vertebral strength of 2 samples per group exceeded the load cell capacity of 475N)). *^a^* Values are significantly different from maternal CON at weaning. *^b^* Values are significantly different from maternal HF at weaning. BD, values below the level of detection, no comparison made. NM, not measured due to small blood volume or bone size from weanlings.

There were no differences in plasma concentrations of leptin, IL-6, 17β-estradiol and progesterone in offspring at weaning and 3 months of age. No comparison was made for TNF-α in offspring at weaning as some rats had values below the level of detection of the assay. However at weaning, more females from dams fed HF diet (n = 3) had detectable levels than females from dams fed CON diet (n = 0). There were no differences in plasma TNF-α of offspring at 3 months of age (Table 3).

#### 2.3.3. Bone Mineral and Strength in Femurs and Lumbar Vertebrae

There was a significant interaction of age and maternal diet for whole femur BMD. Weanling offspring from dams fed HF diet had greater BMD than weanling offspring from dams fed CON diet. Furthermore, weanling offspring from dams fed CON diet had lower whole femur BMD than at 3 months of age, and weanling offspring from dams fed HF diet had lower whole femur BMD than at 3 months of age. There were also significant main effects for age when examining BMC and BMD at all bone sites. Female offspring at 3 months compared to weaning, regardless of maternal diet, had greater BMC and BMD at all bone sites due to growth (Table 3). There were no differences in peak load at the femur midpoint, femur neck or LV3 between groups at 3 months of age (Table 3).

#### 2.3.4. Fatty Acid Composition of Femurs

Femurs of offspring were responsive to maternal diet at weaning and 3 months of age. There were significant interactions for age and maternal diet regarding percent mole fractions of total SFAs, MUFAs, PUFAs and *n*6 PUFAs (Table 4).

**Table 4:** Female offspring femur fatty acid composition.

Fatty Acid	Weaning		3 Months		*P* Values		
	Maternal CON	Maternal HF	Maternal CON	Maternal HF	Age x Diet	Age	Diet
14:0	6.38 ± 0.52	3.15 ± 0.17 *^a^*	1.66 ± 0.06 *^a^*	1.92 ± 0.12 *^b^*	<0.001	<0.001	<0.001
16:0	23.96 ± 0.47	25.53 ± 0.21	22.67 ± 0.62	26.46 ± 1.30	0.178	0.823	0.002
18:0	10.98 ± 0.51	13.16 ± 0.50	7.76 ± 0.23	8.38 ± 0.60	0.115	<0.001	0.007
16:1	2.91 ± 0.17	3.23 ± 0.14	4.98 ± 0.23	5.24 ± 0.37	0.920	<0.001	0.255
18:1	17.44 ± 0.64	30.32 ± 0.66 *^a^*	24.22 ± 0.65 *^a^*	23.69 ± 0.88 *^b^*	<0.001	0.916	<0.001
18:3*n*3	1.72 ± 0.06	0.36 ± 0.02 *^a^*	2.37 ± 0.06 *^a^*	2.05 ± 0.11 *^b,c^*	<0.001	<0.001	<0.001
20:3*n*3	5.79 ± 0.21	5.99 ± 0.31	4.56 ± 0.80	3.42 ± 0.23	0.167	<0.001	0.330
18:2*n*6	19.14 ± 0.30	10.44 ± 0.22 *^a^*	25.94 ± 0.50 *^a^*	23.36 ± 1.00 *^b,c^*	<0.001	<0.001	<0.001
Total SFAs	46.99 ± 0.93	45.20 ± 0.67	33.71 ± 0.78 *^a^*	38.55 ± 1.83 *^b,c^*	0.009	<0.001	0.208
Total MUFAs	21.86 ± 0.63	35.24 ± 0.2 *^a^*	30.23 ± 0.75 *^a^*	30.09 ± 1.02 *^b^*	<0.001	0.056	<0.001
Total PUFAs	31.17 ± 0.61	19.60 ± 0.40 *^a^*	36.06 ± 1.38 *^a^*	31.36 ± 1.08 *^b,c^*	0.002	<0.001	<0.001
*n*3 PUFAs	9.29 ± 0.39	7.20 ± 0.36	7.93 ± 0.76	6.82 ± 0.24	0.324	0.086	0.003
*n*6 PUFAs	21.88 ± 0.30	12.40 ± 0.18 *^a^*	28.13 ± 0.70 *^a^*	24.54 ± 1.07 *^b,c^*	<0.001	<0.001	<0.001

Notes: Data is expressed as mean ± SEM (n = 8–9 per group) for percent mole fraction of femur total fatty acids. Percent mole fraction of fatty acids below 1% are not shown. *^a^* Values are significantly different from maternal CON at weaning. *^b^* Values are significantly different from maternal HF at weaning. *^c^* Values are significantly different from maternal CON at 3 months of age.

At weaning, offspring from dams fed HF diet had higher MUFAs, as well as lower total and *n*6 PUFAs compared to offspring from dams fed CON diet. Offspring from dams fed HF diet at 3 months of age had lower SFAs and MUFAs and higher total and *n*6 PUFAs compared to weanling offspring from dams fed the same diet. Furthermore, offspring from dams fed CON diet at 3 months had lower SFAs and higher MUFAs, total PUFAs, and *n*6 PUFAs than weanling offspring from dams fed the same diet. Three month old offspring from dams fed HF diet had higher SFAs and lower total and *n*6 PUFAs than offspring from dams fed CON diet.

### 2.4. Effect of Maternal Diet in Male Offspring

#### 2.4.1. Body Weight and Body Composition

At 3 months of age, offspring of dams fed HF diet had significantly greater body weight than offspring of dams fed CON diet (Figure 1b). There were significant main effects for age and maternal diet for % lean mass and % fat mass (Table 5).

**Table 5:** Male offspring body composition, plasma hormones, and bone outcomes.

Outcomes	Weaning		3 Months		*P* Values		
	Maternal CON	Maternal HF	Maternal CON	Maternal HF	Age x Diet	Age	Diet
**Body Composition**							
% Lean mass	85.3 ± 2.0	68.5 ± 2.6	64.9 ± 4.5	62.9 ± 4.2	0.063	0.002	0.020
% Fat mass	13.4 ± 1.9	29.5 ± 2.5	33.6 ± 4.4	35.6 ± 4.1	0.070	0.001	0.022
% Bone mass	1.31 ± 0.08	2.00 ± 0.12 *^a^*	1.49 ± 0.09	1.48 ± 0.08 *^b^*	<0.001	0.083	0.001
**Plasma Hormones**							
Leptin (pg/mL)	6255 ± 520	7367 ± 902	5535 ± 916	6796 ± 1126	0.934	0.475	0.193
IL-6 (pg/mL)	179 ± 28	230 ± 50	217 ± 34	188 ± 24	0.264	0.954	0.759
TNF-α (pg/mL)	11.58 ± 1.03	13.29 ± 1.62	11.51 ± 2.92	8.74 ± 0.99	0.275	0.437	0.738
MCP-1 (pg/mL)	470 ± 74	495 ± 142	308 ± 49	274 ± 23	0.721	0.027	0.958
**Bone Mineral**							
*Whole femur*							
BMC (mg)	18.8 ± 1.2	25.3 ± 2.5	499.0 ± 7.3	524.0 ± 8.2	0.121	<0.001	0.011
BMD (mg/mm^2^)	0.35 ± 0.01	0.42 ± 0.03	2.11 ± 0.03	2.13 ± 0.01	0.261	<0.001	0.068
*1/3 Proximal femur*							
BMC (mg)	5.3 ± 0.4	7.0 ± 0.7	172.2 ± 2.9	179.8 ± 3.5	0.227	<0.001	0.060
BMD (mg/mm^2^)	0.36 ± 0.01	0.42 ± 0.03	2.18 ± 0.02	2.20 ± 0.01	0.383	<0.001	0.039
*LV1-3*							
BMC (mg)	10.3 ± 1.1	13.8 ± 1.5	377.9 ± 10.1	394.5 ± 18.4	0.540	<0.001	0.350
BMD (mg/mm^2^)	0.31 ± 0.02	0.36 ± 0.03	2.07 ± 0.02	2.14 ± 0.04	0.571	<0.001	0.029
**Bone Strength**							
*Femur midpoint*							
Peak load (N)	NM	NM	172.2 ± 9.6	174.0 ± 5.3			0.872
*Femur neck*							
Peak load (N)	NM	NM	110.8 ± 11.2	127.3 ± 8.5			0.258
*LV3*							
Peak load (N)	NM	NM	347.1 ± 42.6	444.9 ± 33.0			0.098

Notes: Data is expressed as mean ± SEM ((n = 9 per group); except for measures of body composition at weaning (n = 7/group) and LV3 peak load (n = 4 or 5/group as vertebral strength exceeded the load cell capacity of 475 N)). *^a^* Values are significantly different from maternal CON at weaning. *^b^* Values are significantly different from maternal HF at weaning. NM, not measured due to small bone size of weanlings.

As such, offspring at weaning had greater lean mass and lower fat mass than at 3 months of age, regardless of maternal diet. Also, offspring of dams fed HF diet had lower lean mass and higher fat mass than those of dams fed CON diet, regardless of age. There were significant interactions of age and maternal diet for % bone mass. As such, weanling offspring from dams fed HF diet showed greater bone mass than weanling offspring from dams fed CON diet. Additionally, weanling offspring from dams fed HF diet had greater % bone mass than at 3 months of age (Table 5).

#### 2.4.2. Plasma Hormone Concentrations

There were no differences in plasma leptin, IL-6, TNF-α, or MCP-1 between groups (Table 5).

#### 2.4.3. Bone Mineral and Strength in Femurs and Lumbar Vertebrae

There were significant main effects of age for BMC and BMD at all bone sites. As such, 3 month old offspring had greater BMC and BMD in the whole femur, 1/3 proximal femur, and LV1-3 than offspring at weaning, regardless of maternal diet. There were also significant main effects of maternal diet for whole femur BMC, 1/3 proximal femur BMD, and LV1-3 BMD. As such, offspring from dams fed HF diet had greater whole femur BMC, 1/3 proximal femur BMD, and LV1-3 BMD than offspring from dams fed CON diet, regardless of age (Table 5). Peak load did not differ between males from dams fed HF or CON diet at the femur midpoint, femur neck, or LV3 at 3 months of age (Table 5).

#### 2.4.4. Fatty Acid Composition of Femurs

Femurs from offspring were responsive to maternal diet at weaning but not 3 months of age based on lipid composition. There was a significant main effect of age for SFAs between groups, whereby offspring at weaning had greater SFAs than at 3 months of age, regardless of maternal diet. There were significant interactions for age and maternal diet regarding percent mole fractions of total MUFAs, PUFAs, and *n*6 PUFAs (Table 6). Weanling offspring from dams fed HF diet had greater MUFAs and lower total PUFAs and *n*6 PUFAs than weanling offspring of dams fed CON diet. At weaning, offspring from dams fed HF diet had greater MUFAs and lower total PUFAs and *n*6 PUFAs than at 3 months of age. Also at weaning, all offspring from dams fed CON diet had lower MUFAs than at 3 months of age.

**Table 6:** Male offspring femur fatty acid composition.

Fatty Acid	Weaning		3 Months		*p* Values		
	Maternal CON	Maternal HF	Maternal CON	Maternal HF	Age x Diet	Age	Diet
14:0	6.02 ± 0.32	3.05 ± 0.28 *^a^*	1.92 ± 0.06 *^a^*	2.18 ± 0.11 *^b^*	<0.001	<0.001	<0.001
16:0	25.00 ± 0.45	22.49 ± 1.59	26.66 ± 1.17	28.21 ± 0.85	0.077	0.002	0.666
18:0	11.61 ± 1.47	15.26 ± 1.54	9.39 ± 1.39	8.19 ± 2.33	0.166	0.011	0.479
16:1	3.03 ± 0.22	2.78 ± 0.17	6.03 ± 0.31	6.05 ± 0.40	0.633	<0.001	0.675
18:1	17.31 ± 0.68	24.94 ± 1.95 *^a^*	19.28 ± 1.41	18.69 ± 1.07 *^b^*	0.006	0.131	0.016
18:3*n*3	1.66 ± 0.47	0.33 ± 0.12 *^a^*	1.68 ± 0.07	1.64 ± 0.13 *^b^*	0.022	0.018	0.015
18:2*n*6	16.16 ± 0.67	8.23 ± 0.65 *^a^*	18.22 ± 0.94	18.21 ± 1.41 *^b^*	<0.001	<0.001	<0.001
20:2*n*6	1.16 ± 0.41	2.33 ± 0.96	1.04 ± 0.26	1.35 ± 0.43	0.486	0.366	0.228
20:4*n*6	4.79 ± 1.19	4.25 ± 0.88	5.65 ± 1.45	5.95 ± 1.37	0.734	0.304	0.926
Total SFAs	48.93 ± 1.31	45.44 ± 1.61	40.56 ± 0.98	41.12 ± 1.80	0.178	<0.001	0.326
Total MUFAs	23.61 ± 1.37	35.53 ± 1.77 *^a^*	28.88 ± 1.32 *^a^*	27.41 ± 1.19 *^b^*	<0.001	0.337	0.001
Total PUFAs	27.46 ± 1.68	19.03 ± 0.85 *^a^*	30.56 ± 1.56	31.47 ± 1.51 *^b^*	0.003	<0.001	0.013
*n*3 PUFAs	8.76 ± 1.18	7.34 ± 0.49	10.2 ± 1.02	10.31 ± 0.61	0.390	0.018	0.460
*n*6 PUFAs	18.7 ± 0.68	11.69 ± 1.13 *^a^*	20.36 ± 0.90	21.16 ± 1.06 *^b^*	<0.001	<0.001	0.003

Notes: Data is expressed as mean ± SEM (n = 8 or 9 per group) for percent mole fraction of femur total fatty acids. Percent mole fraction of fatty acids below 1% are not shown. *^a^* Values are significantly different from maternal CON at weaning. *^b^* Values are significantly different from maternal HF at weaning.

## 3. Discussion

Maternal HF feeding did not have long lasting effects on body composition, bone mineral (BMC or BMD), or bone strength in female and male offspring at 3 months of age. Thus, although maternal high fat feeding resulted in some differences in body composition and bone health at weaning, these effects did not persist at young adulthood, suggesting that the intervention did not result in nutritional programming for the outcomes measured. Although differences in fatty acid composition of femurs were evident in weanling male offspring, these differences did not persist into young adulthood, however, female offspring had sustained differences.

Maternal HF feeding influenced offspring body composition and femur bone composition (mineral and fatty acids) at weaning in both male and female offspring. These effects included lower % lean mass and higher % fat and % bone mass in offspring exposed to maternal HF diet compared to maternal CON diet. This finding is consistent with previous literature pertaining to maternal HF feeding and fat and lean mass of offspring – bone mass was not measured in these studies [17,20]. Our study is the first to measure and report % bone mass in weanling offspring exposed to a maternal HF diet. This higher % bone mass may be attributed to the milk composition during suckling. Others have shown that milk obtained from dams fed HF diet reflects the diet consumed, exposing offspring to a similar diet as their mother while suckling [1,17,21]. It is possible that the higher % bone mass is due to the greater content of nutrients that enhance accumulation of bone mineral, including calcium and vitamin D. Similarly, the higher femur BMD of weanling female offspring from dams fed a HF diet may be also due to greater delivery of nutrients that enhanced mineral accumulation in dam’s milk, particularly since the difference in femur BMD was not evident at 3 months of age. The higher plasma level of MCP-1 in female offspring from dams fed HF diet at weaning corresponds with the greater % fat mass at weaning due to a higher fat content of the maternal diet, and possibly the dam’s milk. The finding that MCP-1 is lower at 3 months of age compared to weaning among female offspring of mothers fed a HF diet corresponds with our finding that higher % fat mass at weaning does not persist at 3 months of age, following the consumption of a CON diet.

The fatty acid composition of femurs showed that both males and females are responsive to changes in fatty acid composition of mother’s diet. At weaning, femur bone fatty acid composition reflected the fatty acid composition of femurs from the dams. Moreover, our data show that after consuming CON diet post-weaning, during a rapid period of growth, the fatty acid composition of femurs obtained from 3 month old male offspring reflected the fatty acid content of the CON diet. Thus, maternal diet alone did not permanently alter offspring bone fatty acid composition. Rather, the bone fatty acid composition reflected the diet consumed, in agreement with previous studies that have manipulated the fatty acid composition of diets fed to older rats [3,12]. Moreover, it has been shown that skeletal sites (femurs *versus* lumbar vertebrae) in rats respond similarly with respect to diet-induced changes in fatty acid composition [12,22,23]. Our findings that fatty acid composition was similar between groups in males at 3 months of age, may partly explain why we did not observe differences in peak load—the functional measure of bone health—at either the femur or LV3 at 3 months of age. However, fatty acid composition of femurs from 3 month old female offspring did not reflect diet consumed. This may be because females have a slower growth rate than males, gaining half the body weight of males by 3 months of age. Thus, unlike females, we speculate that male offspring may have used these lipid stores to support their rapid growth. Despite these differences in fatty acid composition, there were no functional differences in femur bone strength. This may suggest that greater femur SFA content does not affect fracture risk at early adulthood.

Since maternal body weight [24,25] and bone health may influence that of offspring, we specifically measured many of the same outcomes in dams that were measured in offspring. Litter characteristics (number of pups, ratio of females to males, litter weight at birth) were similar between groups but differences in litter weight were observed at postnatal age of 12 days, an age at which offspring would still be exclusively suckling and not able to consume mother’s diet. The higher litter weight of the pups from mothers fed HF diet may be explained by differences in milk composition, as discussed earlier, and was not due to heavier body weight at time of breeding; as only final body weight, at the end of the suckling period, resulted in heavier body weight of dams fed HF diet compared to dams fed CON diet.

## 4. Experimental

### 4.1. Animals and Diets

All experimental procedures complied with the Canadian Council on Animal Care [26] and were approved by the Brock University Animal Care Committee. Female Wistar rats (n = 23, Charles River Laboratories, St. Constant, QC, Canada) were obtained at weaning (~28 days old) and were housed two per cage with a 12:12 hour light/dark cycle. Rats were randomly assigned to receive one of two diets: control (CON; AIN93G diet, 7% soybean oil by weight) or high fat (HF; modified AIN93G diet, 20% lard by weight), *ad libitum* for 10 weeks. CON diet contained 18.8% protein, 63.9% carbohydrate, and 17.2% energy from fat (TD.94045; Harlan Teklad, Mississauga, ON, Canada). HF diet contained 19.1% protein, 39.9% carbohydrate, and 41.0% energy from fat (TD.02016; Harlan Teklad). The fatty acid compositions of the two diets, as measured by gas chromatography, are shown in Table 7. The amount of protein, vitamins, and minerals in the HF diet was increased by a factor of 1.2 compared to the CON diet to account for the 1.2 fold higher energy content (3.8 kcal/g CON diet *versus versus* 4.4 kcal/g HF diet). This adjustment was done to ensure that any observed effects in the offspring could be attributed to the higher level of fat in the diet, and not to lower levels of other nutrients on an energy basis. Body weights were recorded weekly (VI-1200 electronic scale, Acculab, Bradford, MA, USA).

After 10 weeks of feeding, females were bred with male Wistar rats (n = 6) of similar age. Each male rat was bred with the same number of females from each intervention, ensuring equal paternal influence among groups. Nine females from each diet group became pregnant during the 1 week breeding period. Female rats were fed their respective diets throughout pregnancy and lactation. Offspring were weaned at postnatal day (PND) 19. Dams were fasted overnight and were euthanized the following morning by an overdose of sodium pentobarbital (12 mg/100 g body weight) and blood and bones (femurs and lumbar vertebrae 1-3; LV1-3) were collected. Two male and two female offspring per litter were euthanized at weaning—one male and one female were measured for body composition while the other male and female from the litter were used for blood and bone collection. Additionally, two males and two females per litter were weaned onto CON diet and studied until 3 months of age. Body weights of offspring were recorded weekly from weaning through to 3 months of age. It is important to note that for every outcome, only one male and one female per litter are reported to ensure there was no “litter effect” [27]. At 3 months of age, similar to weaning, two male and two female offspring per litter were euthanized with an overdose of sodium pentobarbital after an overnight fast. One rat per gender was used for body composition assessment while one rat per gender was used for blood and bone collection. All blood samples and excised bones were stored at −80 °C until analysis.

**Table 7:** Fatty acid composition of treatment diets.

Fatty acid	CON	HF
14:0	0.59 ± 0.01	2.31 ± 0.03
16:0	12.25 ± 0.10	25.79 ± 0.03
18:0	4.19 ± 0.02	11.95 ± 0.21
16:1	0.20 ± 0.01	2.75 ± 0.01
18:1	20.42 ± 0.36	36.34 ± 0.17
18:3*n*3	8.46 ± 0.06	0.82 ± 0.01
18:2*n*6	51.91 ± 0.31	16.38 ± 0.29
Total SFAs	18.52 ± 0.10	41.67 ± 0.12
Total MUFAs	21.01 ± 0.36	40.10 ± 0.21
*n*3 PUFAs	8.46 ± 0.06	1.10 ± 0.01
*n*6 PUFAs	52.00 ± 0.30	17.14 ± 0.29

Values are expressed as percent mole fraction of total fatty acids, percent mole fraction of fatty acids below 1% for both diets are not shown; CON, AIN93G diet; HF, AIN93G with 20% lard by weight; SFA, saturated fatty acid; MUFA, monounsaturated fatty acid; PUFA, polyunsaturated fatty acid.

### 4.2. Body Composition Assessment

Body composition (lean, fat and bone mass) was determined using dual-energy X-ray absorptiometry (DEXA; pSabre, Orthometrix, Naples, FL, USA) and specialized software (Host Software version 3.9.4; Scanner Software version 1.2.0) in offspring at weaning and 3 months of age. All rats were placed on the scanning field in a prone position (nose facing left side of scanning field) prior to analysis. In weanling rats, the region of interest (ROI) surrounded the abdominal region (4 cm in width, 3.5 cm in height), with the lower boundary immediately above the femur heads [28] and scan parameters of 10 mm/s (speed) and a resolution of 0.2 × 0.2 mm. To analyze 3 month old offspring, the ROI was 10 cm in width and 7 cm in height, fixed in the abdominal region, again with the lower boundary immediately above the femur heads [28] and scan parameters of 10 mm/s (speed) and a resolution of 0.5 × 1.0 mm. Percent lean, fat and bone mass were calculated by dividing lean, fat or bone mass in grams by the total mass of the ROI and multiplying by 100.

### 4.3. Collection of Plasma and Hormone Analyses

Blood was collected in EDTA tubes and centrifuged at 3000 RPM for 10 minutes at 4 °C to obtain plasma from dams and offspring (weaning and 3 months of age). Plasma was analyzed using Magpix technology with xPONENT software (Luminex Corporation, Austin, TX, USA). Plasma from dams and all offspring were measured for leptin, interleukin-6 (IL-6), monocyte chemoattractant protein-1 (MCP-1), and tumor necrosis factor-alpha (TNF-α) using a Rat Metabolic Magnetic Bead Panel (Cat. #RMHMAG-84K; Millipore Corporation, Billerica, MA, USA). Plasma from dams and 3 month old female offspring were measured for 17β-estradiol and progesterone using a Steroid/Thyroid Hormone Magnetic Bead Panel (Cat. # STTHMAG-21K; Millipore Corporation).

### 4.4. Bone Mineral and Strength of Excised Femurs and Lumbar Vertebrae

Femur and LV1-3 BMC (mg) and BMD (mg/mm^2^) of dams and offspring at weaning and 3 months of age were measured using DEXA (pSabre, Orthometrix) and specialized software (host software version 3.9.4; scanner software version 1.2.0) [3]. Scans of femurs and LV1-3 were conducted using the following parameters: speed of 2 mm/s, resolution of 0.1 × 0.1 mm (weanlings); or speed of 10 mm/s, resolution of 0.2 × 0.2 mm (dams, 3 month old offspring). Because DEXA does not measure potential structural differences, bone strength—specifically peak load—was measured as it is influenced by both bone mineral content and structure. To measure bone strength, peak load was measured at the femur midpoint, femur neck, and 3rd lumbar vertebrae (LV3) in dams and 3 month old offspring using a materials testing system (Model 4442, Instron, Norwood, MA, USA) and specialized software (Series IX Automated Materials Tester, version 8.1 5.00, Instron, Norwood, MA, USA). A cross-head was lowered perpendicularly to the sample at a constant speed of 2 mm/min, until peak load was achieved (*i.e.*, fracture).

### 4.5. Femur Lipid Analysis

Femurs obtained from dams and offspring were sliced in half using a band saw. Bone marrow was discarded and fragments of bone were wrapped in aluminum foil and placed in liquid nitrogen. The bones were hammered and then pulverized using a mortar and pestle under liquid nitrogen as previously described [12]. The resultant powder was added into 15 mL glass screw cap Kimax tubes and total lipids were extracted with 2:1 chloroform:methanol [29]. Extracted lipids were dried under nitrogen, weighed, and reconstituted in 2 mL of chloroform to form the lipid stock.

Total lipid from each sample was saponified and methylated as previously described [12]. Briefly, 0.0625 mg of lipid from each lipid stock was saponified and methylated at 100 °C using 0.5 M of potassium hydroxide and 6% H_2_SO_4_-MeOH, respectively. The resulting fatty acid methyl esters were separated on a UFM-RTX WAX analytical column (Thermo Electron Corp., Milan, Italy) using gas chromatography (Trace GC Ultra, Thermo Electron Corp.) fitted with a fast flame ionization detector, a split-splitless injector, and Triplus AS autosampler [30]. Fatty acids were identified by comparison of retention times with those of a known standard (Supelco 37 component FAME mix, Supelco, Bellefonte, PA, USA) and absolute amounts of individual fatty acids were calculated with the aid of an internal standard, tridecanoic acid (13:0), added to the samples prior to methylation. Prior analysis determined no detectable endogenous 13:0 in the samples (data not shown).

### 4.6. Statistical Analyses

Statistical analyses were performed using SigmaStat (version 3.5, Systat, Chicago, IL, USA). For dams, independent samples t-tests were used for body weight (at breeding and 6 months of age), plasma hormones, bone mineral, bone strength, and bone lipid profile. For offspring, repeated measures 2-way ANOVA followed by a Student-Newman Keul’s *post-hoc* analysis was used to analyze changes in body weight. The two factors used were age (1 month, 2 months, and 3 months of age) and maternal diet (CON, HF). Offspring body composition (lean, fat and bone mass expressed as % of ROI), plasma hormones (leptin, IL-6, MCP-1, TNF-α), bone mineral of excised femurs and lumbar vertebrae, and femur lipid profile were conducted using 2-way ANOVAs. The two factors used were age (weaning and 3 months of age) and maternal diet (CON, HF). Independent samples t-tests were used to assess 17β-estradiol and progesterone as there was only sufficient quantity of sample for measurement at 3 months of age in females. Independent samples t-tests were also used to analyze bone strength of offspring at 3 months of age; bones at weaning age were too small for the strength analyses. For all offspring analyses, males and females were assessed separately because of established differences in growth and bone development. Data are expressed as mean ± SEM. Significance was determined as *p* ≤ 0.05.

## 5. Conclusions

In summary, the findings from this study using Wistar rats demonstrate that early developmental differences in body composition and composition of femurs and lumbar vertebra mediated by maternal high fat feeding were not sustained in offspring provided a healthy post-weaning diet.

## References

[1] Franco J.G., Fernandes T.P., Rocha C.P.D., Calvino C., Pazos-Moura C.C., Lisboa P.C., Moura E.G., Trevenzoli I.H. (2012). Maternal high-fat diet induces obesity and adrenal and thyroid dysfunction in male rat offspring at weaning. J. Physiol. (London).

[2] Xiao Y., Cui J., Li Y.X., Shi Y.H., Wang B., Le G.W., Wang Z.P. (2011). Dyslipidemic high-fat diet affects adversely bone metabolism in mice associated with impaired antioxidant capacity. Nutrition.

[3] Lau B.Y., Fajardo V.A., McMeekin L., Sacco S.M., Ward W.E., Roy B.D., Peters S.J., LeBlanc P.J. (2010). Influence of high-fat diet from differential dietary sources on bone mineral density, bone strength, and bone fatty acid composition in rats. Appl. Physiol. Nutr. Metab..

[4] Cao J.J., Gregoire B.R., Gao H.W. (2009). High-fat diet decreases cancellous bone mass but has no effect on cortical bone mass in the tibia in mice. Bone.

[5] Ward W.E., Kim S., Robert Bruce W. (2003). A western-style diet reduces bone mass and biomechanical bone strength to a greater extent in male compared with female rats during development. Br. J Nutr..

[6] Parhami F., Tintut Y., Beamer W.G., Gharavi N., Goodman W., Demer L.L. (2001). Atherogenic high-fat diet reduces bone mineralization in mice. J. Bone Miner. Res..

[7] Xiao Y., Cui J., Shi Y.H., Sun J., Wang Z.P., Le G.W. (2010). Effects of duodenal redox status on calcium absorption and related genes expression in high-fat diet-fed mice. Nutrition.

[8] Lac G., Cavalie H., Ebal E., Michaux O. (2008). Effects of a high fat diet on bone of growing rats. Correlations between visceral fat, adiponectin and bone mass density. Lipids Health Dis..

[9] Macri E.V., Gonzales Chaves M.M., Rodriguez P.N., Mandalunis P., Zeni S., Lifshitz F., Friedman S.M. (2012). High-fat diets affect energy and bone metabolism in growing rats. Eur. J. Nutr..

[10] Posey K.A., Clegg D.J., Printz R.L., Byun J., Morton G.J., Vivekanandan-Giri A., Pennathur S., Baskin D.G., Heinecke J.W., Woods S.C. (2009). Hypothalamic proinflammatory lipid accumulation, inflammation, and insulin resistance in rats fed a high-fat diet. Am. J. Physiol. Endocrinol. Metab..

[11] Howie G.J., Sloboda D.M., Kamal T., Vickers M.H. (2009). Maternal nutritional history predicts obesity in adult offspring independent of postnatal diet. J. Physiol..

[12] Sacco S.M., Jiang J.M., Reza-Lopez S., Ma D.W., Thompson L.U., Ward W.E. (2009). Flaxseed combined with low-dose estrogen therapy preserves bone tissue in ovariectomized rats. Menopause.

[13] Reinwald S., Li Y., Moriguchi T., Salem N., Watkins B.A. (2004). Repletion with (n-3) fatty acids reverses bone structural deficits in (n-3)-deficient rats. J. Nutr..

[14] Li Y., Seifert M.F., Lim S.Y., Salem N., Watkins B.A. (2010). Bone mineral content is positively correlated to n-3 fatty acids in the femur of growing rats. Brit. J. Nutr..

[15] Watkins B.A., Li Y., Allen K.G., Hoffmann W.E., Seifert M.F. (2000). Dietary ratio of (n-6)/(n-3) polyunsaturated fatty acids alters the fatty acid composition of bone compartments and biomarkers of bone formation in rats. J. Nutr..

[16] Krasnow S.M., Nguyen M.L., Marks D.L. (2011). Increased maternal fat consumption during pregnancy alters body composition in neonatal mice. Am. J. Physiol. Endocrinol. Metab..

[17] Sun B., Purcell R.H., Terrillion C.E., Yan J., Moran T.H., Tamashiro K.L. (2012). Maternal high-fat diet during gestation or suckling differentially affects offspring leptin sensitivity and obesity. Diabetes.

[18] Lanham S.A., Roberts C., Hollingworth T., Sreekumar R., Elahi M.M., Cagampang F.R., Hanson M.A., Oreffo R.O. (2010). Maternal high-fat diet: Effects on offspring bone structure. Osteoporos. Int..

[19] Devlin M.J., Grasemann C., Cloutier A.M., Louis L., Alm C., Palmert M.R., Bouxsein M.L. (2013). Maternal perinatal diet induces developmental programming of bone architecture. J. Endocrinol..

[20] White C.L., Purpera M.N., Morrison C.D. (2009). Maternal obesity is necessary for programming effect of high-fat diet on offspring. Am. J. Physiol. Regul. Integr. Comp. Physiol..

[21] Purcell R.H., Sun B., Pass L.L., Power M.L., Moran T.H., Tamashiro K.L. (2011). Maternal stress and high-fat diet effect on maternal behavior, milk composition, and pup ingestive behavior. Physiol. Behav..

[22] Lau B.Y., Ward W.E., Kang J.X., Ma D.W. (2009). Vertebrae of developing fat-1 mice have greater strength and lower n-6/n-3 fatty acid ratio. Exp. Biol. Med..

[23] Lau B.Y., Ward W.E., Kang J.X., Ma D.W. (2009). Femur epa and dha are correlated with femur biomechanical strength in young fat-1 mice. J. Nutr. Biochem..

[24] Whitaker R.C., Wright J.A., Pepe M.S., Seidel K.D., Dietz W.H. (1997). Predicting obesity in young adulthood from childhood and parental obesity. N. Engl. J. Med..

[25] Bayol S.A., Farrington S.J., Stickland N.C. (2007). A maternal ‘junk food’ diet in pregnancy and lactation promotes an exacerbated taste for “junk food” and a greater propensity for obesity in rat offspring. Br. J. Nutr..

[26] Olfert E.D., Cross B.M., McWilliam A.A. (1993). Guide to the Care and Use of Experimental Animals.

[27] Wainwright P.E. (1998). Issues of design and analysis relating to the use of multiparous species in developmental nutritional studies. J. Nutr..

[28] Jiang J.M.Y., Sacco S.M., Ward W.E. (2008). Ovariectomy-induced hyperphagia does not modulate bone mineral density or bone strength in rats. J. Nutr..

[29] Folch J., Lees M., Sloane Stanley G.H. (1957). A simple method for the isolation and purification of total lipides from animal tissues. J. Biol. Chem..

[30] Bradley N.S., Heigenhauser G.J.F., Roy B.D., Staples E.M., Inglis J.G., LeBlanc P.J., Peters S.J. (2008). The acute effects of differential dietary fatty acids on human skeletal muscle pyruvate dehydrogenase activity. J. Appl. Physiol..

